# Pharmacological Modulation of the Short-Lasting Effects of Antagonistic Direct Current-Stimulation Over the Human Motor Cortex

**DOI:** 10.3389/fpsyt.2012.00067

**Published:** 2012-07-05

**Authors:** Leila Chaieb, A. Antal, D. Terney, W. Paulus

**Affiliations:** ^1^Department of Clinical Neurophysiology, Georg-August University of GöttingenGöttingen, Germany; ^2^Danish Epilepsy CentreDianalund, Denmark

**Keywords:** human, tDCS, neuroplasticity, d-cycloserine, pergolide, motor cortex

## Abstract

Combined administration of transcranial direct current-stimulation (tDCS) with either pergolide (PER) or d-cycloserine (d-CYC) can prolong the excitability-diminishing effects of cathodal, or the excitability enhancing effect of anodal stimulation for up to 24 h poststimulation. However, it remains unclear whether the potentiation of the observed aftereffects is dominated just by the polarity and duration of the stimulation, or the dual application of combined stimulation and drug administration. The present study looks at whether the aftereffects of oral administration of PER (a D1/D2 agonist) or d-CYC (a partial NMDA receptor agonist), in conjunction with the short-duration antagonistic application of tDCS (either 5 min cathodal followed immediately by 5 min anodal or vice versa), that alone only induces short-lasting aftereffects, can modulate cortical excitability in healthy human subjects, as revealed by a single-pulse MEP (motor-evoked-potential) paradigm. Results indicate that the antagonistic application of tDCS induces short-term neuroplastic aftereffects that are dependent upon the order of the application of short-duration stimulation. The administration of d-CYC resulted in a marked inhibition of cortical excitability under the application of tDCS in both stimulation orders. Intake of PER resulted in an increase in cortical excitability in both stimulation orientations, but was non-significant compared to the placebo condition. These results indicate that the aftereffects of tDCS are dependent upon the order of stimulation applied, and also demonstrate the prolongation of tDCS aftereffects when combined with the administration of CNS active drugs.

## Introduction

The effect that transcranial direct current-stimulation (tDCS) exerts on the intact human cortex is closely related to the modulation of cortical excitability and neuronal activity, which are key mechanisms for learning and memory processing (Paulus, 2004). The relevant stimulation parameters encompass the polarity, the current strength, size of the stimulated area, and duration of the stimulation (Nitsche et al., 2008; Stagg and Nitsche, 2011) and are considered to be safe as assessed by several studies (Nitsche et al., 2003b; Iyer et al., 2005; Poreisz et al., 2007). The most common way to evaluate changes in cortical excitability is by applying transcranial magnetic stimulation (TMS) to the motor cortex, since it allows the measurement of reproducible and quantifiable effects through the analysis of motor-evoked-potentials (MEPs). Anodal stimulation increases the amplitude of MEPs while cathodal stimulation decreases them (Nitsche and Paulus, 2000, 2001). The primary effect of tDCS is a neuronal de- or hyperpolarization of the membrane potential (Creutzfeldt et al., 1962; Bindman et al., 1964), whereby the induced aftereffects depend on *N*-methyl-d-aspartate (NMDA) receptor-efficacy changes (Liebetanz et al., 2002). Studies investigating the combined administration of pharmacological agents combined with tDCS, has provided valuable insights into the mechanisms and modes of action that tDCS exerts on neuronal tissue (for a review, see Nitsche, 2005).

The anatomical structure of the cortex means that when using currents to polarize neuronal tissue in the brain, a homogeneous induction of either excitability increase or decrease is prevented by the folded cortex. If for example, current flows through a cortical gyrus, on one side of the gyrus wall an excitability increase is induced, whereas on the opposing side, a excitability diminution cannot be avoided (Creutzfeldt et al., 1962; Lang et al., 2005; Datta et al., 2009). This is a consideration when utilizing tDCS as treatment in neurological disorders, such as epilepsy, as any unwanted excitability increases may theoretically worsen seizure frequency or intensity. The aim of the present study was to find a pharmacological solution for this problem by investigating whether we were able to induce changes in cortical excitability using short-duration antagonistic applications of tDCS in combination with CNS active drugs that have been shown to prolong neuroplasticity-inducing aftereffects.

To investigate this question, we stimulated the motor cortex in two opposing directions during one stimulation application in addition to administering d-cycloserine (d-CYC) a drug selectively prolonging the excitability enhancement induced by anodal stimulation) or pergolide (PER; a drug selectively prolonging the excitability reduction induced by cathodal stimulation) as well as a placebo (PLC) control. After drug intake, a 5-min anodal followed by a 5-min cathodal (or vice versa) antagonistic stimulation was applied to the primary motor cortex (M1) in a healthy participant population. Previous studies proved that 5 min stimulation duration alone did not evoke aftereffects lasting longer than 5 min (Nitsche and Paulus, 2000). A study by Nitsche et al. (2004a) showed that d-CYC, a partial NMDA agonist, selectively potentiated the duration of motor-cortical excitability enhancements induced by anodal tDCS from approximately 1 h up to 24 h poststimulation without affecting cathodal inhibition. In contrast, PER, a combined D1/D2 receptor agonist, prolonged the excitability-diminishing effects of cathodal tDCS for up to 24 h after stimulation (Nitsche et al., 2006).

d-CYC was initially introduced as a tuberculostatic agent (Walker and Murdoch, 1957) and was later found to be a CNS active drug at very low doses. d-CYC acts at the glycine-binding site of the NMDA receptor, thus facilitating the opening of the NMDA channel (Thomas et al., 1988). In slice preparations, it has been shown that the activation of this subunit is of importance for inducing long-term potentiation (LTP) effects, and that d-CYC can enhance LTP-like neuroplastic effects (Watanabe et al., 1992).

PER, a D1/D2 dopamine receptor agonist, has been shown to have effects on intracortical excitability in the human motor cortex, where it enhanced intracortical inhibition (Ziemann et al., 1996). A more recent study showed that PER consolidated excitability decreases generated by applications of tDCS to the M1, up until the morning after stimulation. Furthermore, co-administration of PER and sulpiride, allowing for D1 activation in the presence of D2 receptor blocking, was not able to re-establish the characteristic alterations in cortical excitability induced by transcranial direct currents. In another study, PER was shown to enhance the effect of tDCS (or in particular cathodal tDCS) in reducing the amplitude of laser-evoked pain potentials applied over the human M1 (Terney et al., 2008). In this study, PER was administered to 12 healthy subjects before tDCS, after assessing subjective acute pain perception induced by a Tm:YAG laser. The amplitudes of the N2 component of the laser-evoked pain potentials, as well as the subjective rating scores, were significantly reduced up to 2 h poststimulation, with PER increasing the efficacy of the effect of the cathodal stimulation for up to 24 h poststimulation. These data strongly argue for the importance of D2 receptor activity for the induction of increases and decreases in prolonged NMDA receptor dependent motor-cortical excitability shifts in humans, as well as a role in the induction of neuroplastic effects in the intact human cortex.

The hypothesis central to this exploratory study was to pursue whether in a paradigm of antagonistic tDCS current flow direction, the choice of either drug shown to potentiate neuroplastic effects in the cortex will finally determine the direction of tDCS aftereffects, either toward an excitation or inhibition of cortical excitability.

## Materials and Methods

### Subjects

Eight healthy subjects participated in each experiment (six male; mean age 25.5). All gave written informed consent. The study was approved by the ethics committee of the University of Göttingen, and conformed to the Declaration of Helsinki.

### Current-stimulation of the motor cortex

Direct currents were transferred via a pair of saline-soaked surface sponge electrodes (35 cm^2^) fixed to the scalp and delivered by a specially developed battery-driven constant current stimulator (NeuroConn, Ilmenau, Germany). The motor-cortical electrode was placed over the representational field of the right abductor digiti minimi muscle (ADM) as identified by TMS, and the other electrode was located contralateral to the right orbit. In the different experiments, the currents flowed continuously for 10 min (5 min anodal + 5 min cathodal or vice versa) with an intensity of 1.0 mA.

### Pharmacological interventions

d-CYC (100 mg), PER (0.025 mg; combined with 10 mg domperidone to avoid nausea) or equivalent placebo (PLC) drugs were administered to the subjects orally 2 h prior to the onset of stimulation. By these means, the *verum* drugs were able to induce a stable plasma level (Deleu et al., 2002) and produce prominent effects in the CNS (Nitsche et al., 2004a, 2006; Kuo et al., 2008). To avoid interference of plasticity induction by cumulative drug effects, each experimental session was separated by at least 1 week. Both the subjects and the investigator conducting the experiment were blinded as to the respective pharmacological and stimulation conditions administered during each experimental session.

### Measurement of motor-cortical excitability

To detect current-driven changes of excitability, motor-evoked potentials (MEPs) of the right ADM were recorded following stimulation of its motor-cortical representational field by single-pulse TMS. These were induced using a Magstim 200 magnetic stimulator (Magstim, Whiteland, Dyfed, UK) and a figure-of-eight magnetic coil (diameter of one winding = 70 mm; peak magnetic field = 2.2 T). The coil was held tangentially to the skull, with the handle pointing backward and laterally at 45° from the midline. The optimal position was defined as the site where stimulation resulted consistently in the largest MEP. Surface EMG was recorded from the right ADM by use of Ag–AgCl electrodes in a belly tendon montage. Raw signals were amplified, band-pass filtered (2–3 kHz; sampling rate, 5 kHz), digitized with a micro 1401 AD converter (Cambridge Electronic Design, Cambridge, UK) controlled by Signal Software (Cambridge Electronic Design, version 2.13), and stored on a personal computer for offline analysis. The intensity of the stimulator output was adjusted for baseline recording so that the average stimulus led to an MEP of ~1 mV.

### Experimental procedures

The experiments were conducted in a randomized, repeated measurement design. The subjects were seated in a reclining chair. First, the left motor-cortical representational field of the right ADM was identified by use of TMS (coil position that leads to the largest MEPs of ADM). Then one DC stimulation electrode, to which in the following the terms cathodal or anodal stimulation refer, was fixed at this position, and the other one was fixed at the contralateral forehead above the orbit.

A baseline of TMS-evoked MEPs (60 stimuli) was recorded at 0.25 Hz. Afterward, anodal, cathodal, or sham tDCS was performed for 10 min. After termination of tDCS, 60 MEPs were recorded at 0.25 Hz 0, 5, and then every 10 min up to 60 min poststimulation.

### Statistical analysis

MEP amplitude means were calculated for each time bin covering baseline (60 stimuli) and poststimulation time-points (60 stimuli). These were normalized and are given as ratios of the pre-current baselines.

Separate repeated measurement anovas [independent variables time course, current-stimulation (TYPE: anodal-cathodal or anodal-cathodal), drug condition (PLC vs. d-CYC or PPER), dependent variable MEP amplitude) were calculated for each time bin up to 60 min post-tDCS, for the different stimulation conditions separately. Student’s *t*-tests (paired samples, two-tailed, level of significance <0.05) were performed at each time point to determine whether the MEP amplitudes differed with regard to placebo or the drug administration.

## Results

None of the subjects reported any adverse events during and after the experiments.

### Placebo administration

**Figure 1:**
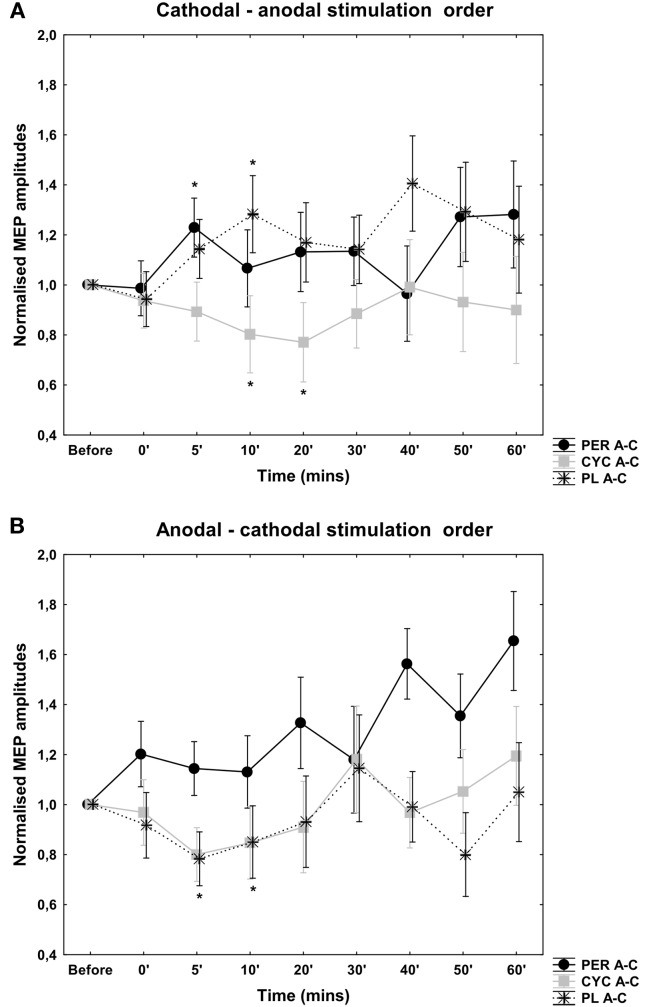
**Effects of antagonistic cathodal-anodal (A) and anodal-cathodal (B) tDCS in conjunction with PER, CYC, and PLC control**. MEP amplitudes are given as mV, vertical bars indicate SEM. With regard to the PLC condition the order of each short-duration antagonistic tDCS application is the predominating factor in modulating short-duration tDCS aftereffects. A tendency toward excitability enhancement was seen after PER administration in the cathodal-anodal stimulation order. After anodal-cathodal tDCS an increase in cortical excitability is also evident, but was not significant. d-CYC administration resulted in a decrease in cortical excitability regardless of the orientation of stimulation.

The cortical excitability change depended on the application of the second type of stimulation: the anodal-cathodal stimulation resulted in a decrease in cortical excitability while the cathodal-anodal stimulation produced excitation (Figures 1A,B). The ANOVA revealed no significant main effect with regard to the TYPE of stimulation [*F*(1, 14) = 1.46, *p* = 0.24] and time course [*F*(7, 98) = 0.91, *p* = 0.5] but the interaction of the TYPE of stimulation × time course showed a tendency [*F*(7, 98) = 1.8; *p* = 0.06]. Student’s *t*-test showed a significant difference at 5′ and 10′ poststimulation between the anodal-cathodal and cathodal-anodal stimulation conditions (*p* < 0.05, *t* = 2.54; 2.4).

### Cathodal-anodal current direction

After d-CYC administration there was a decrease in cortical excitability using the cathodal-anodal stimulation order (Figure 1). Compared to the PLC stimulation, the anova revealed significant main effect of stimulation [*F*(1, 14) = 5.97, *p* = 0.03], but the time course [*F*(7, 98) = 0.86, *p* = 0.53] and the interaction of stimulation × time course [*F*(7, 98) = 0.89, *p* = 0.51] were not significant. The Student *t*-test showed a significant excitability decrease at 10, and 20 min poststimulation compared to placebo intake (*p* < 0.05, *t* = 3.13; 2.46).

After PER administration there was no detectable after-effect when the cathodal-anodal stimulation combination was applied. Generally, the MEP amplitudes were more unstable compared to the other stimulation condition. Compared to the PLC administration, the anova revealed no main effect of stimulation [*F*(1, 14) = 1.98, *p* = 0.16] and time course [*F*(7, 98) = 1.02, *p* = 0.41] and no significant interaction of stimulation × time course [*F*(7, 98) = 1.14, *p* = 0.32].

### Anodal-cathodal current direction

However, after d-CYC administration there was no significant change in cortical excitability using the anodal-cathodal stimulation order. There was no significant main effect of stimulation [*F*(1, 14) = 0.19, *p* = 0.66] and time course [*F*(7, 98) = 1.54, *p* = 0.16]. The interaction between the type of stimulation and time course was not significant [*F*(7, 98) = 0.43, *p* = 0.8; Figure 1].

Similarly, after PER administration there was no significant aftereffect when the anodal-cathodal stimulation combination was applied. Generally, the MEP amplitudes were more unstable compared to the other stimulation condition. Compared to the PLC administration, the anova revealed no main effect of stimulation [*F*(1, 14) = 0.19, *p* = 0.82] and time course [*F*(7, 98) = 1.43, *p* = 0.19] and no significant interaction of stimulation × time course [*F*(7, 98) = 0.67, *p* = 0.79].

## Discussion

This investigation into the antagonistic application of tDCS revealed that the order of each tDCS primarily determines the induced aftereffects; which was either an increase or decrease in cortical excitability. Initially our hypothesis was to examine whether the administration of a pharmacological agent known to modulate neuroplastic effects in the cortex, would influence the direction of induced aftereffects and/or prolong any measureable aftereffects. We observed that there was no excitatory aftereffect after d-CYC administration for both the cathodal-anodal and anodal-cathodal stimulation orders (only an inhibition was observed), and no inhibitory after-effect after PER administration, irrespective of the current flow direction sequence. A prolongation of tDCS aftereffects outlasting the stimulation duration of 5 min in each polarity (anodal or cathodal tDCS) was observed. In a previous study, Nitsche and Paulus (2000) demonstrated that short-duration tDCS (5 and 7 min) did not produce aftereffects lasting longer than between 5 and 30 min (depending upon the stimulation duration), whereas the present study reports that 10 min of antagonistic tDCS produces at least a 10-min aftereffect post-drug administration. In addition, we observed that the order of stimulation was the dominant modulator of the tDCS-induced aftereffect. For example, when 5 min cathodal-anodal stimulation was applied over the M1, a net excitatory effect was observed, and vice versa for the reverse stimulation combinations for the PLC condition. For the cathodal-anodal stimulation order, there was a net decrease in cortical excitability under d-CYC which showed a significant interaction between the order of stimulation across the measured timecourse (at 5 and 10 min) poststimulation. The administration of PER showed an overall increase in MEP amplitudes but was not significant compared to the PLC condition. In the anodal-cathodal stimulation order, d-CYC and the PLC showed a decrease in cortical excitability over time but this tendency was not significant. PER increased MEP amplitudes compared to the PLC condition, but this was also not significant. The MEPs recorded under PER administration in both stimulation orders were largely unstable and so a significant net increase or decrease in the levels of cortical excitability was difficult to determine. The mechanism of tDCS action has been investigated in many previous human and animal studies (Bindman et al., 1964; Nitsche, 2005; Nitsche et al., 2005) and has also been well characterized by the use of CNS active drugs (Nitsche, 2005). During tDCS, the effects of both anodal and cathodal stimulation are dependent upon fluctuations in membrane potential. However, the induction of tDCS aftereffects can also depend upon synaptic modulation and affect intracortical neurons (as anodal and cathodal DC currents do not influence motor threshold values; Nitsche et al., 2005). The aftereffects of anodal and cathodal tDCS are influenced by the potentiation of receptors at the glutamatergic synapse (Nitsche et al., 2003a), and studies have also shown that they are modulated by dopamine, acetylcholine, and serotonin receptors (Kuo et al., 2007; Monte-Silva et al., 2009; Nitsche et al., 2009). Anodal tDCS is also strongly influenced by GABAergic neurotransmission via the activity of interneurons (Nitsche et al., 2004b). The relatively weak effects of antagonistic tDCS that we have observed in this study may arise for a number of different reasons; the balance between the potentiation of D1 and D2 receptors by PER and the low dosage administered may account for the unstable trend toward cortical excitation. Sampling a larger subject group could also have reduced the high variability in the MEP data and also have made this trend significant. Secondly, the effects of tDCS alone showed that the order of each stimulation application influenced that outcome of the aftereffects. As tDCS is duration dependent, it is also possible that the stimulation duration was not long enough to induce enduring aftereffects. The interaction between PER, d-CYC, and tDCS at the membrane would also have affected the stability of the aftereffects, as tDCS-induced neuroplasticity is NMDA receptor dependent (Liebetanz et al., 2002) and influenced by the ratio of D1/D2 receptors; the potentiation of NMDA receptors may not have been strong enough to overcome the effects of the short-lasting DC modulations. In summary, tDCS aftereffects are dependent upon the polarity of stimulation, duration, and intensity, but are also heavily influenced by neuromodulators potentiating receptors that are present upon the neuronal membrane. Therefore, it is difficult point out a single mode of action that is responsible for the aftereffects observed here.

The effects of antagonistic tDCS have not been widely investigated until now. Priori et al. (1998), whilst looking at DC polarization of the motor cortex, reported that pulses of anodal DC only changed the amplitude of elicited MEPs when there was an alternation in the application of anodal and cathodal DC. Based upon animal data (Stafstrom et al., 1984), they suggested that there may be a degree of neuronal adaptation, whereby neuronal elements compensate for the DC-induced changes in the membrane potential. This indicated that by alternating the anodal-cathodal DC sequence, the targeted neuronal elements were prevented from adapting to the polarizing DC stimulation.

We were able to observe a significant inhibition in cortical excitability when tDCS in the cathodal-anodal current direction was applied under d-CYC administration. These results are similar to the data published by Kuo et al. (2008). The combined application of cathodal-anodal tDCS and d-CYC within independent experimental sessions, were examined in conjunction with mechanisms of homeostatic plasticity during a motor learning task. The excitability diminution induced by cathodal tDCS prior to motor learning, or an excitability enhancement induced by anodal tDCS if combined with the partial NMDA receptor agonist d-CYC, impaired learning performance. Similarly to these data, we have observed a decrease in MEP amplitude, when the cathodal-anodal stimulation condition was applied. However, the stimulation duration was shorter compared to those applied in previous studies with d-CYC, which may account for the differences reported in this study.

In our study, the oral administration of PER did not induce any significant aftereffect poststimulation, and the results were not significantly different from those obtained under the PLC condition although a tendency toward excitability enhancement could be seen. This was possibly due to the increased variability of MEP amplitudes after PER intake. We have seen that administration of PER increased the instability of MEPs (and thus the variability of MEP amplitudes) after the antagonistic administration of tDCS. The possible cause of this instability could be attributed to the very low dose of PER given in this study, compared to those of other studies reporting the effects of PER on transcranial stimulation measured using TMS-elicited MEPs (Lang et al., 2008). As previously mentioned, the variability may also have been decreased by increasing the number of participants that were involved in the study.

Dopaminergic (DA) mechanisms have been demonstrated to stabilize these processes involved in neuroplasticity induction (for a review examining the effects of dopamine on cortical excitability, see Nitsche et al., 2010). DA acting on D1 receptors increases NMDA currents (Cepeda and Levine, 1998). In addition, the enhancement of D2 – and to a lesser degree – of D1 receptors by pergolide consolidated tDCS generated excitability diminution up until the morning post stimulation (Nitsche et al., 2006). A number of recent studies have looked at the interaction of the dopaminergic system with tDCS applied to the cortex. One study examining the dose-dependent effects of dopamine on plasticity processes employed two varying methods to either induce focal (paired-associative stimulation, PAS) or non-focal (tDCS paradigms) plasticity in the motor cortex. The authors demonstrated that administration of varying dosages of ropinirole, a D2/D3 dopamine agonist, resulted in an inverted “U”-shaped dose-response curve on excitability enhancing tDCS and PAS protocols, as well as inhibitory tDCS protocols. They concluded that in high or low dosages, ropinirole attenuated plasticity-inducing processes, and that neuroplasticity processes involving D2 receptor potentiation are subject to dose-dependent effects, and can be considered when examining inhibitory and facilitatory mechanisms of plasticity, depending upon the type of plasticity induced (Monte-Silva et al., 2009). A similar study showed that l-dopa administered in high (200 mg) or low (25 mg) doses abolished facilitatory and inhibitory cortical plasticity, whereas a medium dose (100 mg) reversed facilitation into inhibition in the motor cortex, as well as prolonging inhibitory plasticity (Monte-Silva et al., 2010). Investigations into the impact of dopamine on learning and memory formation demonstrate that dopamine also has a focusing effect on neuroplasticity processes. A study looking at the influence of D1 receptors showed that the balance between D1 and D2 receptor activity is crucial in both the consolidation of resultant excitability changes arising from non-focal (tDCS) and focal (PAS; in this case inhibitory, iPAS, or excitatory, ePAS), neuroplasticity-inducing paradigms, and to generate a focusing effect of plasticity (Nitsche et al., 2009). The possible mechanisms of DC-induced aftereffects were investigated in several previous studies; pharmacological intervention suggests that the aftereffect is *N*-methyl-d-aspartate (NMDA)-receptor dependent (Liebetanz et al., 2002; Nitsche et al., 2004a,b,c). NMDA receptor and intracellular sigma 1 receptor blocker dextromethorphan intake prevented both anodal and cathodal tDCS-induced aftereffects, demonstrating that dextromethorphan critically interferes with the functionality of tDCS irrespective of the polarity of the DC stimulation. It is known that long-lasting NMDA receptor dependent cortical excitability and activity shifts are involved in neuroplastic modification. Another study revealed that NMDA receptor antagonist dextromethorphan did not changes levels of cortical excitability during a short-duration of tDCS, and also prevented any enduring aftereffects of tDCS, independent of stimulation polarity (Nitsche et al., 2003a).

Homeostatic mechanisms are might also play a role in the observable aftereffects of tDCS, a state whereby neurons in the nervous system dynamically adjust synaptic strengths favoring the direction that promotes stability in growing networks. This process is not unlike Hebbian mechanisms of plasticity, but differs fundamentally in the sense that Hebbian mechanisms tend to destabilize neural circuits and homeostatic mechanisms can relate complex neural networks responsible for processes ranging from memory storage to activity dependent development, and so by their very nature are more stable (Turrigiano and Nelson, 2000, 2004). Preconditioning the M1 with tDCS can shape the magnitude and direction of excitability changes induced by a subsequent session of repetitive TMS (rTMS). Lang et al. (2004) published a study demonstrating that anodal tDCS causes a subsequent application of 1 Hz rTMS to reduce corticospinal excitability, while preconditioning with cathodal tDCS induces the reverse effect. However, our present data are not in agreement with these previous results, suggesting that for the manifestation of homeostatic mechanisms longer stimulation durations may be required, or that this kind of plasticity has a limited influence when more components (drug application and antagonistic external stimulation) are administered at the same time.

In this exploratory investigation, we are able to reveal that stimulation duration has a much greater impact on modulating cortical excitability than the administration of sub-therapeutic levels of CNS active drugs, in combination with tDCS. Further experimental work would need to be conducted in order to understand whether it was initially the use of a shorter stimulation duration in this antagonistic stimulation sequence, or the antagonistic administration of tDCS in combination with PER or d-CYC that may have resulted in the aftereffects that we report here; this may be the critical limiting step within the paradigm that we have chosen to implement in this study. Previous studies have highlighted the efficacy of non-invasive brain stimulation devices used in combination both with and without CNS drugs in the treatment of neurological disorders, for example in the treatment of chronic pain (Antal and Paulus, 2011), migraine (Antal et al., 2011), and depression (Loo et al., 2012). With this study we aimed to investigate whether the antagonistic application of tDCS in combination with PER and d-CYC could induce changes in cortical excitability, and modulate these excitability changes long enough to provide an insight into whether short-duration tDCS could be used as a therapeutic approach for neurological disturbances.

## Conclusion

The present study reports that administration of CNS active drugs in combination with short-duration tDCS can modulate tDCS-induced aftereffects in the healthy human motor cortex. The predominant factor influencing the outcome of these effects is the order of antagonistic short-duration tDCS application.

## Conflict of Interest Statement

The authors declare that the research was conducted in the absence of any commercial or financial relationships that could be construed as a potential conflict of interest.

## References

[B1] Antal A. Kriener N. Lang N. Boros K. Paulus W. (2011). Cathodal transcranial direct current stimulation of the visual cortex in the prophylactic treatment of migraine. Cephalalgia 31, 820–828 10.1177/0333102410379889 21398419

[B2] Antal A. Paulus W. (2011). A case of refractory orofacial pain treated by transcranial direct current stimulation applied over hand motor area in combination with NMDA agonist drug intake. Brain Stimul. 4, 117–121 10.1016/j.brs.2010.09.003 21511214

[B3] Bindman L. Lippold O. Redfearn J. W. (1964). The action of brief polarizing currents on the cerebral cortex of the rat (1) during current flow and (2) in the production of long-lasting after-effects. J. Physiol. (Lond.) 172, 369–382 14199369 10.1113/jphysiol.1964.sp007425 PMC1368854

[B4] Cepeda C. Levine M. S. (1998). Dopamine and N-methyl-D-aspartate receptor interactions in the neostriatum. Dev. Neurosci. 20, 1–18 10.1159/000017294 9600386

[B5] Creutzfeldt O. D. Fromm G. H. Kapp H. (1962). Influence of transcortical d-c currents on cortical neuronal activity. Exp. Neurol. 5, 436–452 10.1016/0014-4886(62)90056-0 13882165

[B6] Datta A. Bansal V. Diaz J. Patel J. Reato D. Bikson M. (2009). Gyri-precise head model of transcranial direct current stimulation: improved spatial focality using a ring electrode versus conventional rectangular pad. Brain Stimul. 2, 201–207 10.1016/j.brs.2009.03.005 PMC2790295 20648973

[B7] Deleu D. Northway M. G. Hanssens Y. (2002). Clinical pharmacokinetic and pharmacodynamic properties of drugs used in the treatment of Parkinson’s disease. Clin. Pharmacokinet. 41, 261–309 10.2165/00003088-200241040-00003 11978145

[B8] Iyer M. Mattu U. Grafman J. Lomarev M. Sato S. Wassermann E. M. (2005). Safety and cognitive effect of frontal DC brain polarization in healthy individuals. Neurology 64, 872–875 10.1212/01.WNL.0000152986.07469.E9 15753425

[B9] Kuo M. Unger M. Liebetanz D. Lang N. Tergau F. Paulus W. Nitsche M. A. (2008). Limited impact of homeostatic plasticity on motor learning in humans. Neuropsychologia 46, 2122–2128 10.1016/j.neuropsychologia.2008.02.018 18394661

[B10] Kuo M.-F. Grosch J. Fregni F. Paulus W. Nitsche M. A. (2007). Focusing effect of acetylcholine on neuroplasticity in the human motor cortex. J. Neurosci. 27, 14442–14447 10.1523/JNEUROSCI.2391-07.2007 18160652 PMC6673455

[B11] Lang N. Siebner H. Ernst D. Nitsche M. A. Paulus W. Lemon R. N. Rothwell J. C. (2004). Preconditioning with transcranial direct current stimulation sensitizes the motor cortex to rapid-rate transcranial magnetic stimulation and controls the direction of after-effects. Biol. Psychiatry 56, 634–639 10.1016/j.biopsych.2004.07.017 15522246

[B12] Lang N. Siebner H. Ward N. S. Lee L. Nitsche M. A. Paulus W. Rothwell J. C. Lemon R. N. Frackowiak R. S. (2005). How does transcranial DC stimulation of the primary motor cortex alter regional neuronal activity in the human brain? Eur. J. Neurosci. 22, 495–504 10.1111/j.1460-9568.2005.04233.x 16045502 PMC3717512

[B13] Lang N. Speck S. Harms J. Rothkegel H. Paulus W. Sommer M. (2008). Dopaminergic potentiation of rTMS-induced motor cortex inhibition. Biol. Psychiatry 63, 231–233 10.1016/j.biopsych.2007.04.033 17604004

[B14] Liebetanz D. Nitsche M. Tergau F. Paulus W. (2002). Pharmacological approach to the mechanisms of transcranial DC-stimulation-induced after-effects of human motor cortex excitability. Brain 125(Pt 10), 2238–2247 10.1093/brain/awf238 12244081

[B15] Loo C. K. Alonzo A. Martin D. Mitchell P. B. Galvez V. Sachdev P. (2012). Transcranial direct current stimulation for depression: 3-week, randomised, sham-controlled trial. Br. J. Psychiatry 200, 52–59 10.1192/bjp.bp.111.097634 22215866

[B16] Monte-Silva K. Kuo M. F. Thirugnanasambandam N. Liebetanz D. Paulus W. Nitsche M. A. (2009). Dose-dependent inverted U-shaped effect of dopamine (D2-like) receptor activation on focal and nonfocal plasticity in humans. J. Neurosci. 29, 6124–6131 10.1523/JNEUROSCI.0728-09.2009 19439590 PMC6665507

[B17] Monte-Silva K. Liebetanz D. Grundey J. Paulus W. Nitsche M. A. (2010). Dosage-dependent non-linear effect of L-dopa on human motor cortex plasticity. J. Physiol. (Lond.) 588(Pt 18), 3415–3424 10.1113/jphysiol.2010.190181 20660568 PMC2988508

[B18] Nitsche M. (2005). Pharmacological characterisation and modulation of neuroplasticity in humans. Curr. Neuropharmacol. 3, 217–229 10.2174/1570159054368268

[B19] Nitsche M. Fricke K. Henschke U. Schlitterlau A. Liebetanz D. Lang N. Henning S. Tergau F. Paulus W. (2003a). Pharmacological modulation of cortical excitability shifts induced by transcranial direct current stimulation in humans. J. Physiol. (Lond.) 553(Pt 1), 293–301 10.1113/jphysiol.2003.049916 12949224 PMC2343495

[B20] Nitsche M. Liebetanz D. Lang N. Antal A. Tergau F. Paulus W. (2003b). Safety criteria for transcranial direct current stimulation (tDCS) in humans. Clin. Neurophysiol. 114, 2220–2222; author reply 2222–2223 14580622 10.1016/s1388-2457(03)00235-9

[B21] Nitsche M. Jaussi W. Liebetanz D. Lang N. Tergau F. Paulus W. (2004a). Consolidation of human motor cortical neuroplasticity by D-cycloserine. Neuropsychopharmacology 29, 1573–1578 10.1038/sj.npp.1300517 15199378

[B22] Nitsche M. Liebetanz D. Schlitterlau A. Henschke U. Fricke K. Frommann K. Lang N. Henning S. Paulus W. Tergau F. (2004b). GABAergic modulation of DC stimulation-induced motor cortex excitability shifts in humans. Eur. J. Neurosci. 19, 2720–2726 10.1111/j.0953-816X.2004.03398.x 15147306

[B23] Nitsche M. A. Grundey J. Liebetanz D. Lang N. Tergau F. Paulus W. (2004c). Catecholaminergic consolidation of motor cortical neuroplasticity in humans. Cereb. Cortex 14, 1240–1245 10.1093/cercor/bhh085 15142961

[B24] Nitsche M. Lampe C. Antal A. Liebetanz D. Lang N. Tergau F. Paulus W. (2006). Dopaminergic modulation of long-lasting direct current-induced cortical excitability changes in the human motor cortex. Eur. J. Neurosci. 23, 1651–1657 10.1111/j.1460-9568.2006.04676.x 16553629

[B25] Nitsche M. Paulus W. (2000). Excitability changes induced in the human motor cortex by weak transcranial direct current stimulation. J. Physiol. (Lond.) 527(Pt 3), 633–639 10.1111/j.1469-7793.2000.t01-1-00633.x 10990547 PMC2270099

[B26] Nitsche M. Paulus W. (2001). Sustained excitability elevations induced by transcranial DC motor cortex stimulation in humans. Neurology 57, 1899–1901 10.1212/WNL.57.10.1899 11723286

[B27] Nitsche M. Seeber A. Frommann K. Klein C. C. Rochford C. Nitsche M. S. Fricke K. Liebetanz D. Lang N. Antal A. Paulus W. Tergau F. (2005). Modulating parameters of excitability during and after transcranial direct current stimulation of the human motor cortex. J. Physiol. 568, 291–303 10.1113/jphysiol.2005.092429 16002441 PMC1474757

[B28] Nitsche M. A. Cohen L. G. Wassermann E. M. Priori A. Lang N. Antal A. Paulus W. Hummel F. Boggio P. S. Fregni F. Pascual-Leone A. (2008). Transcranial direct current stimulation: state of the art 2008. Brain Stimul. 1, 206–223 10.1016/j.brs.2008.06.004 20633386

[B29] Nitsche M. A. Kuo M.-F. Grosch J. Bergner C. Monte-Silva K. Paulus W. (2009). D1-receptor impact on neuroplasticity in humans. J. Neurosci. 29, 2648–2653 10.1523/JNEUROSCI.5366-08.2009 19244540 PMC6666237

[B30] Nitsche M. A. Monte-Silva K. Kuo M. F. Paulus W. (2010). Dopaminergic impact on cortical excitability in humans. Rev. Neurosci. 21, 289–298 10.1515/REVNEURO.2010.21.4.289 21086761

[B31] Paulus W. (2004). Outlasting excitability shifts induced by direct current stimulation of the human brain. Suppl. Clin. Neurophysiol. 57, 708–714 10.1016/S1567-424X(09)70411-8 16106673

[B32] Poreisz C. Boros K. Antal A. Paulus W. (2007). Safety aspects of transcranial direct current stimulation concerning healthy subjects and patients. Brain Res. Bull. 72, 208–214 10.1016/j.brainresbull.2007.01.004 17452283

[B33] Priori A. Berardelli A. Rona S. Accornero N. Manfredi M. (1998). Polarization of the human motor cortex through the scalp. Neuroreport 9, 2257–2260 10.1097/00001756-199807130-00020 9694210

[B34] Stafstrom C. E. Schwindt P. C. Crill W. E. (1984). Cable properties of layer V neurons from cat sensorimotor cortex in vitro. J. Neurophysiol. 52, 278–289 6481433 10.1152/jn.1984.52.2.278

[B35] Stagg C. J. Nitsche M. A. (2011). Physiological basis of transcranial direct current stimulation. Neuroscientist 17, 37–53 10.1177/1073858410386614 21343407

[B36] Terney D. Bergmann I. Poreisz C. Chaieb L. Boros K. Nitsche M. A. Paulus W. Antal A. (2008). Pergolide increases the efficacy of cathodal direct current stimulation to reduce the amplitude of laser-evoked potentials in humans. J. Pain Symptom Manage. 36, 79–91 10.1016/j.jpainsymman.2007.08.014 18358692

[B37] Thomas J. W. Hood W. F. Monahan J. B. Contreras P. C. O’Donohue T. L. (1988). Glycine modulation of the phencyclidine binding site in mammalian brain. Brain Res. 442, 396–398 10.1016/0006-8993(88)91533-8 2836022

[B38] Turrigiano G. G. Nelson S. B. (2000). Hebb and homeostasis in neuronal plasticity. Curr. Opin. Neurobiol. 10, 358–364 10.1016/S0959-4388(00)00091-X 10851171

[B39] Turrigiano G. G. Nelson S. B. (2004). Homeostatic plasticity in the developing nervous system. Nat. Rev. Neurosci. 5, 97–107 10.1038/nrn1327 14735113

[B40] Walker W. C. Murdoch J. M. (1957). Cycloserine in the treatment of pulmonary tuberculosis: a report on toxicity. Tubercle 38, 297–302 10.1016/S0041-3879(57)80179-2 13496234

[B41] Watanabe Y. Himi T. Saito H. Abe K. (1992). Involvement of glycine site associated with the NMDA receptor in hippocampal long-term potentiation and acquisition of spatial memory in rats. Brain Res. 582, 58–64 10.1016/0006-8993(92)90316-2 1386772

[B42] Ziemann U. Bruns D. Paulus W. (1996). Enhancement of human motor cortex inhibition by the dopamine receptor agonist pergolide: evidence from transcranial magnetic stimulation. Neurosci. Lett. 208, 187–190 10.1016/0304-3940(96)12575-1 8733301

